# Properties of Graphene/Shape Memory Thermoplastic Polyurethane Composites Actuating by Various Methods

**DOI:** 10.3390/ma7031520

**Published:** 2014-02-27

**Authors:** Jin Ho Park, Trung Dung Dao, Hyung-il Lee, Han Mo Jeong, Byung Kyu Kim

**Affiliations:** 1 Department of Chemistry, Energy Harvest-Storage Research Center, University of Ulsan, Ulsan 680-749, Korea; E-Mails: dino1875@naver.com (J.H.P.); daotrungdung_vn@yahoo.com (T.D.D.); sims0904@ulsan.ac.kr (H.L.); 2 Department of Polymer Science and Engineering, Pusan National University, Busan 609-735, Korea; E-Mail: bkkim@pnu.edu

**Keywords:** shape memory polyurethane, graphene, composite, direct heating, resistive heating, IR heating

## Abstract

Shape memory behavior of crystalline shape memory polyurethane (SPU) reinforced with graphene, which utilizes melting temperature as a shape recovery temperature, was examined with various external actuating stimuli such as direct heating, resistive heating, and infrared (IR) heating. Compatibility of graphene with crystalline SPU was adjusted by altering the structure of the hard segment of the SPU, by changing the structure of the graphene, and by changing the preparation method of the graphene/SPU composite. The SPU made of aromatic 4,4′-diphenylmethane diisocyanate (MSPU) exhibited better compatibility with graphene, having an aromatic structure, compared to that made of the aliphatic hexamethylene diisocyanate. The finely dispersed graphene effectively reinforced MSPU, improved shape recovery of MSPU, and served effectively as a filler, triggering shape recovery by resistive or IR heating. Compatibility was enhanced when the graphene was modified with methanol. This improved shape recovery by direct heating, but worsened the conductivity of the composite, and consequently the efficiency of resistive heating for shape recovery also declined. Graphene modified with methanol was more effective than pristine graphene in terms of shape recovery by IR heating.

## 1. Introduction

Shape memory polymer is used as a temperature sensing intelligent material in a wide variety of fields, such as biomedical science, textile engineering, and robotics [1]. It is relatively cheap, light, easily manufactured, and has large recoverable strain compared to shape memory alloys. It generally consists of two phases, a fixed structure for memorizing the original shape and a thermally reversible phase which conforms to a transient shape. Crystalline or glassy phase, chain entanglement, or crosslinking are used as the fixed structure. The thermally reversible phase is designed to have a large drop in elastic modulus upon heating above the shape recovery temperature (*T_s_*). Therefore, the glass transition temperature (*T_g_*) of the amorphous phase and the melting temperature (*T_m_*) of the crystalline phase are used as a *T_s_* [2–4].

Thermoplastic polyurethane, which has a microphase-separated structure due to the incompatibility between hard and soft segments, can exhibit shape memory effects, because hard and soft segments serve as a fixed structure and as a reversible phase, respectively. Tailor-made properties of thermoplastic polyurethane can be obtained using well-designed combinations of monomeric materials, because a wide range of monomeric materials are commercially available. Therefore, *T_s_* and the mechanical properties of the shape memory polyurethane (SPU) can be tailored to specific applications [5,6].

Shape recovery and the recovery force of shape memory polymers can be improved by incorporating nanocarbons, such as graphene, carbon nanotubes, or carbon nanofibers [7–10]. In addition, shape memory polymers reinforced by nanocarbon can be triggered not only by direct heating but also by IR heating or by resistive heating which facilitates remote activation [3,11–14].

Graphene has emerged as an exciting, new nanocarbon, because it has novel properties: an electrical conductivity of 10^6^ S/cm, a basal plane elastic modulus of 1 TPa, a thermal conductivity of 5000 W/m·K, a high aspect ratio, and a large specific surface area of 2600 m^2^/g [15,16]. Bulk quantities of graphene can be produced effectively by the thermal exfoliation of graphite oxide (GO) in which the GO sheets are reduced and exfoliated simultaneously upon rapid heating due to the thermal decomposition of oxygen-containing groups of GO and the pressure of gas products (mainly CO_2_) that builds up instantaneously at the gallery between the sheets [17]. This method is economical and eco-friendly because it does not use any solvents during the reduction process. Thermally-exfoliated graphene is normally comprised of few-layer graphene sheets with specific surface areas ranging from 400 to 1500 m^2^/g according to Brunauer, Emmett, and Teller (BET) measurements using nitrogen adsorption in the dry state [17–19]. The advantages in productivity and simplicity of thermal exfoliation make the approach promising for many applications in which flat and perfect single-layer graphene is not mandatory and quantity is more important. Because some oxygen-containing functional groups such as epoxy or hydroxyl groups remain on the graphene even after thermal reduction [20], utilization of these remnant functional groups for chemical modifications or functionalization can upgrade graphene’s performance in various applications.

The unique properties of nanofiller/polymer composites stem not only from the inherent properties of the nanofillers, but also from optimized interfacial contact and interactions between the nanofiller and polymer matrix. In our previous papers, we reported that the shape memory performance of SPU was improved by the incorporation of graphene and the improvement was enhanced when the graphene was modified to have hydroxyl groups on the surface, which formed covalent bonds with SPU during *in situ* preparation of the graphene/SPU composites [21,22]. The SPUs used in the study were amorphous ones utilizing *T_g_* as a *T_s_*.

In nanofiller/crystalline polymer composites, the crystallization behavior of the matrix polymer is affected by the nanofiller. The nanofiller enhances the crystallization because it can serve as a nucleus for crystallization. However, in some cases, the interaction between the nanofiller and matrix polymer can inhibit the chain rearrangement for crystallization [23,24]. Therefore, the compatibility between graphene and SPU can affect the properties of the graphene/SPU composite in complicated ways when the SPU is crystalline and one utilizes *T_m_* as a *T_s_*.

In this study, compatibility between graphene and crystalline thermoplastic SPU was fine-tuned by functionalization of the graphene, by changing the structure of the hard segment in the SPU, and by using different composite preparation methods. The effects of these changes on the performance of the shape memory composites were examined. In addition, the functionality of graphene not only modulates the compatibility but also alters the electric conductivity and IR absorption efficiency. Therefore, to collect deployable data to optimize the properties of the shape memory composites, shape recovery was triggered using various external stimuli such as direct heating, resistive heating, and IR heating.

## 2. Results and Discussion

### 2.1. Characteristics of Graphene/SPU Composites

**Figure 1.**
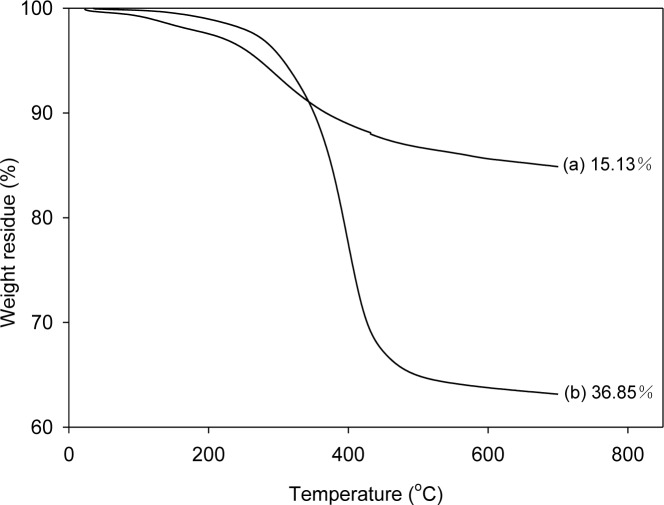
Thermogravimetric analysis (TGA) thermogram of graphene separated from (**a**) MSPU-M-MG10 and (**b**) MSPU-S-MG10.

In order to analyze the amount of SPU attached to graphene, the graphenes were separated from the composites, which were prepared with 1 part graphene per 100 parts of SPU (1 phr), and were analyzed by thermogravimetric analysis (TGA). To separate the graphene from graphene/SPU composite, the composite was dissolved in a 300-fold amount of dimethylformamide (DMF), and then the suspended graphenes were separated by filtration. The filtered graphenes were thoroughly washed with DMF in a Soxhlet extractor for three days and dried under vacuum at 80 °C for one day for TGA. The weight losses for pristine graphene (PG) and modified graphene (MG) at 700 °C were only 1.62% and 3.41%, respectively [21]. Whereas the SPU prepared with 4,4′-diphenylmethane diisocyanate (MDI) (MSPU) and the SPU prepared with hexamethylene diisocyanate (HDI) (HSPU) were mostly pyrolyzed at 700 °C; the weight losses were 97.87% and 99.77%, respectively. Figure 1 shows that the weight loss at 700 °C of the graphene separated from MSPU-S-MG10 (the sample names of composites are designated at experimental details) is 36.85%, whereas that of the graphene separated from MSPU-P-MG10 is only 15.13%. The difference, 21.72%, indicates that the MSPU attached to the graphene increased when the composite was prepared by *in situ* methods due to the grafting on MG during *in situ* preparation. However, the weight loss of the graphene separated from MSPU-S-PG10 was 15.71%, and that of MSPU-P-PG10 was 14.97%. This small difference in weight loss shows that the functional groups on PG on to which SPU can graft during *in situ* preparation are not so much as those on MG. The weight loss of the graphenes separated from HSPU-S-MG10 (48.17 wt%) was also much higher than that of the graphene separated from HSPU-P-MG10 (38.28 wt%); The difference is 9.89%. This also demonstrates that HSPU grafted to graphene much more when prepared by the *in situ* method.

**Figure 2.**
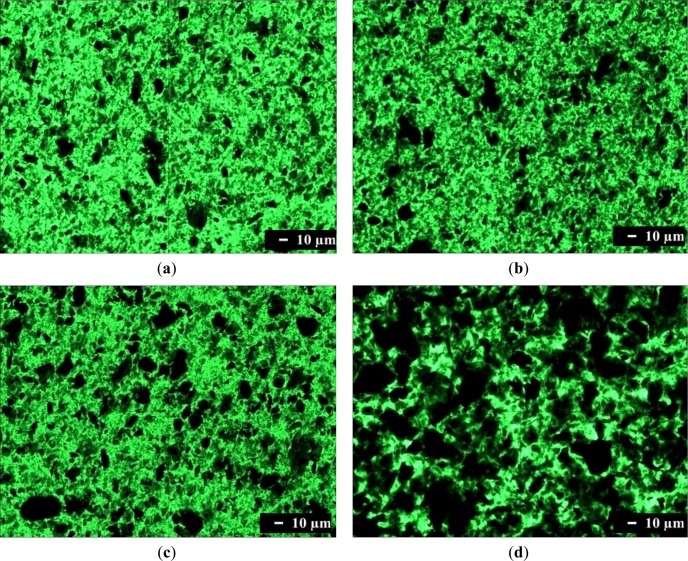
Optical microscopy images of composites: (**a**) MSPU-S-MG10; (**b**) MSPU-S-PG10; (**c**) MSPU-P-MG10; and (**d**) HSPU-S-MG10.

Optical microscopy was used to characterize the dispersion of graphene in a composite on the micrometer scale. Optical microscopy images of composites containing 1.0 phr of graphene are shown in Figure 2. Single-layer graphene is transparent because it absorbs only 2.3% of the light intensity, independent of the wavelength of light. Therefore, single-layer graphene cannot be observed readily with an optical microscope. However, transparency decreases when light hits many graphenes while passing through a material, or when graphenes are stacked into multi-layers or agglomerated. Figure 2a shows that translucent or opaque graphenes are dispersed finely in the SPU matrix, although some are agglomerated. The number of black aggregates is increased in Figure 2b, and further in Figure 2c. These results show that graphene with more surface functional groups, and the composite preparation by the *in situ* method rather than by the physical mixing method, are better for fine dispersion of graphene in the MSPU matrix. This demonstrates that graphene grafted with a higher amount of SPU can disperse better in SPU matrix due to the compatibility of the grafted SPU and graphene. The presence of large amounts of black aggregated graphenes in Figure 2d shows that HSPU has poor dispersion characteristics compared with MSPU, which demonstrates that graphene, which has an aromatic structure, can disperse better in a polyurethane with an aromatic segment than with an aliphatic segment.

**Figure 3.**
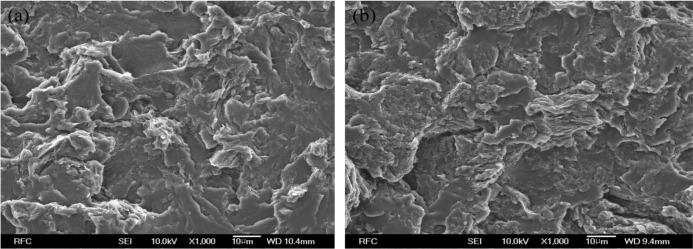
Scanning electron microscope (SEM) images of composites: (**a**) HSPU-S-MG20 and (**b**) MSPU-S-MG20.

The scanning electron microscope (SEM) image of HSPU-S-MG20 (Figure 3a) shows large, heterogeneous particles of aggregated graphenes. However, the fractured surface of MSPU-S-MG20 (Figure 3b) is relatively smooth and it is hard to find large particles of aggregated graphenes in Figure 3b. These SEM images also demonstrate that MG disperses more finely in MSPU than in HSPU.

Table 1 shows that the conductivity of MSPU-S-PG10 is about 10^7^-fold enhanced compared with that of MSPU, indicating that 1.0 phr of PG is well dispersed and forms an effective, interconnected conductive network in the MSPU matrix. However, the conductivity increase was reduced when MG was used instead of PG (Table 1). In addition, the composites of MSPU prepared by the *in situ* method exhibit lower conductivity compared to those prepared by the physical mixing method at the same graphene content (Table 1). Conductivity is improved when the contact between conductive fillers is intimate in the aggregated conductive channel [25]. Increased grafting limits the contact between dispersed graphenes, and consequently, limits the conductivity enhancement [26]. This is the reason for the lower conductivity. In Table 1, the HSPU composites generally exhibit lower conductivity compared with those of MSPU. Poor dispersion of graphene in the HSPU matrix, as observed in Figure 2, is a cause of this smaller increase in conductivity.

**Figure 4.**
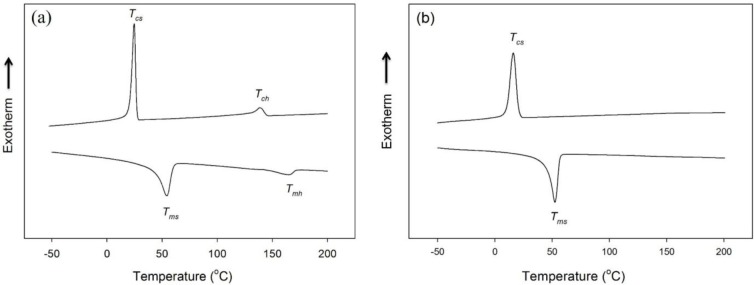
Differential scanning calorimetry (DSC) thermograms of (**a**) HSPU and (**b**) MSPU obtained on cooling (upper) and subsequent heating (lower) scans.

The differential scanning calorimetry (DSC) thermograms of HSPU obtained from the cooling scan and the subsequent heating scan exhibited two separate crystallization and melting peaks of the polycaprolactone (PCL) soft segment and hard segment (Figure 4a). In contrast, the DSC thermograms of MSPU exhibited a crystallization peak and a melting peak of the PCL soft segment only (Figure 4b). The thermal properties of SPU and their composites, the crystallization temperatures of soft and hard segments (*T_cs_* and *T_ch_*), their heats of crystallization (*ΔH_cs_* and *ΔH_ch_*), the melting temperatures of soft and hard segments (*T_ms_* and *T_mh_*) and their heats of fusion (*ΔH_ms_* and *ΔH_mh_*) are summarized in Table 2. In the composites, the crystallization of the matrix polymer can be improved by the nucleating effect of the dispersed filler. However, the interaction between the filler and matrix polymer can hinder rearrangements needed for crystallization of the matrix polymer [27,28]. In the PG/MSPU composites prepared by the *in situ* method, *T_cs_*, *ΔH_cs_*, and *ΔH_ms_* were all higher than those of pristine MSPU (Table 2), indicating that the crystallization of soft PCL segments was enhanced by the nucleating effect of PG. However, for MSPU-P-PG10 prepared by the physical mixing method, the *T_cs_*, *ΔH_cs_*, and *ΔH_ms_* values were all smaller than those of pristine MSPU, suggesting that the hindrance effect is dominant when dispersed poorly. In the MG/MSPU composites prepared by the *in situ* method, *T_cs_*, *ΔH_cs_*, and *ΔH_ms_* were all increased compared with those of pristine MSPU at low MG content. However, they become marginal or negative at high MG content (2.0 phr) (Table 2). This demonstrates that the interaction between MG and SPU, which inhibits the crystallization of SPU, becomes evident at high MG content because the amount of MSPU grafted on MG is high. In the case of MG/HSPU composites prepared by the *in situ* method, the *T_cs_*, *ΔH_cs_*, *ΔH_ms_*, values and the *T_ch_*, *ΔH_ch_*, *ΔH_mh_* values were all lower than those of pristine HSPU (Table 2). These results demonstrate that the hindrance effect rather than the nucleation effect by MG is dominant in these MG/HSPU composites. In short, the data in Table 2 show that the crystallization enhancement due to the nucleating effect of small amounts of dispersed graphenes is present when the graphenes are finely dispersed.

**Figure 5.**
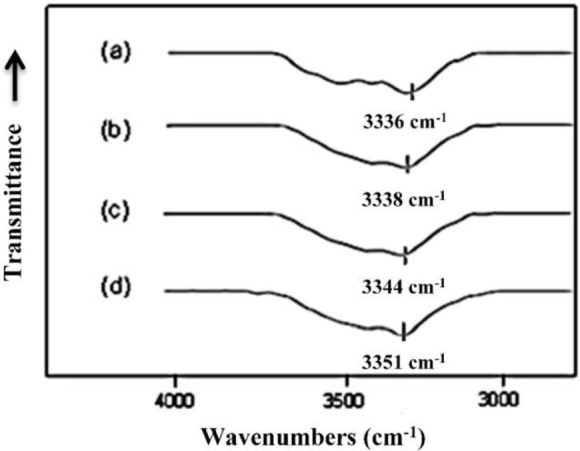
Fourier transform infrared (FTIR) spectra, in the region between 3000 and 3800 cm^−1^, of (**a**) MSPU; (**b**) MSPU-S-MG05; (**c**) MSPU-S-MG10; and (**d**) MSPU-S-MG20.

The free N–H and hydrogen bonded N–H groups of polyurethanes have characteristic Fourier transform infrared (FTIR) absorption bands due to the stretching vibration motion at 3450 and 3330 cm^−1^, respectively [29]. As a result, the N–H stretching vibration band in the 3300 cm^−1^ usually shows broad complex features and is very sensitive to the hydrogen-bonding distribution in polyurethanes [29]. The IR absorption peak in this region moves to higher wavenumber when the hydrogen bond is reduced, as observed by Brunette *et al*. [30]. The FTIR spectra, in the region between 3000 and 3800 cm^−1^, of MSPU and its composites prepared by the *in situ* method with MG are presented in Figure 5. The FTIR spectrum of MSPU (Figure 5a) exhibits a peak of N–H stretching vibration at 3336 cm^−1^ and this peak position moves to higher wavenumber as the content of MG in the composite is increased, indicating that MG is well dispersed in the hard segment domain and thus the hydrogen bond between hard segments of MSPU is disturbed, as we observed in graphene/waterborne polyurethane composites [31]. In the case of the MG/HSPU composites prepared by the *in situ* method or the PG/MSPU composites prepared by physical mixing method, the peak position changes were marginal (not shown in this paper), suggesting that graphene and the hard segment were not intimately mixed. These results show that the compatibility of MG, which has an aromatic structure, is better with aromatic MDI-based hard segments compared with aliphatic HDI-based hard segments, and the compatibility of MG and MDI-based hard segments is improved when the composite is prepared by the *in situ* method rather than by the physical method, as observed in Figures 2 and 3. The broad shoulder around 3500 cm^−1^ may be attributed to the O-H groups at the polyurethane chain end and on graphene.

Table 1 shows that the densities of graphene/SPU composites are less than that of SPU itself. Considering that the density of graphene is similar to that of pure graphite (2.25 g/cm^3^) [32] and is higher than that of SPU, this result suggests that an additional free volume was created in the composite. In the nanofiller-polymer composite, the equilibrium packing density of the matrix polymer at the interface is lower than the bulk due to entropic effects [33]. This free volume increase at the interface is a cause of the density decrease observed for graphene/SPU composites (Table 1). In addition, because the equilibrium packing at 25 °C is denser than that at 60 °C, the polymer chains should rearrange to a dense equilibrium packing on cooling from 60 to 25 °C after casting. However, the physical interactions or chemical bonds between graphene and matrix SPU molecules restrict the rearrangement necessary for equilibrium packing. This is another reason for the density decrease observed in graphene/SPU composites. Table 1 shows that the density decrease by graphene is more evident when composites were prepared by the *in situ* method rather than by physical mixing, and when MG was used instead of PG. These results demonstrate that higher degree of covalent bond formation occurs between the matrix polymer and graphene during the *in situ* preparation when graphene has more hydroxyl groups present, and the enhanced covalent bonds increase the restrictions against equilibrium packing which results in less dense packing.

### 2.2. Tensile Properties and Shape Memory Behavior of Graphene/SPU Composites

Tensile properties of SPU and their composites measured at 60 °C are shown in Table 3. In the PG/MSPU composites prepared by the *in situ* method, the secant modulus at 100% elongation (SM) increases noticeably as the PG content in the composites is increased. However, for MG/MSPU composites prepared by the *in situ* method, this increase of SM is almost saturated at 0.5 phr of MG. This shows that the well-ordered hard segment structure that optimizes hydrogen bonding was scattered by intimately mixed MG as observed in FTIR spectra (Figure 5). Consequently, reinforcement by the hard segment was also weakened at high MG content. The looser packing that was demonstrated in the density data is another reason for the marginal enhancement of the reinforcing effect at high MG content. In the case of MG/HSPU composites made by the *in situ* method, the SM increased more than 2-fold from that of HSPU by 0.5 phr MG. However it decreased again with higher MG content. The looser packing and the decreased crystallinity of the hard segment at higher MG content (Table 2) caused this decreased SM.

**Figure 6.**
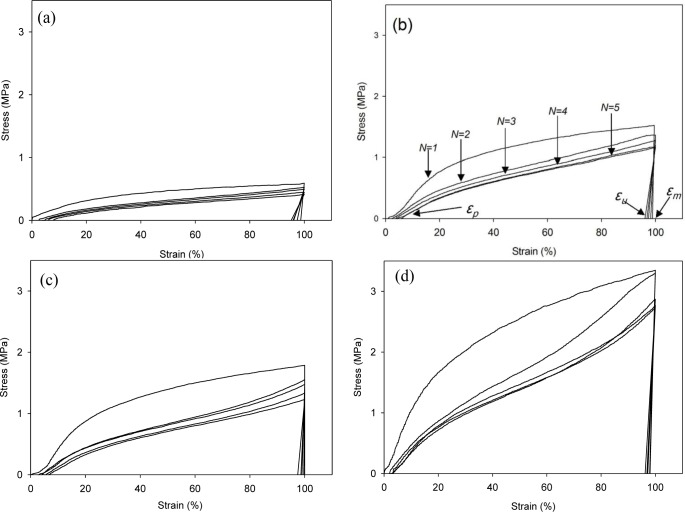
Shape memory behavior of (**a**) MSPU; (**b**) MSPU-S-PG05; (**c**) MSPU-S-PG10; and (**d**) MSPU-S-PG20.

The cyclic tests to examine the shape memory effect of PG/MSPU composite films by direct heating are shown in Figure 6, and their results are summarized in Table 4. Shape recovery and fixity in Table 4 were defined by the Equations (1) and (2):

$$\text{Shape recovery}(\%)=\frac{\varepsilon_{m}-\varepsilon_{p}}{\varepsilon_{m}}\times 100$$ (1)

$$\text{Shape fixity}(\%)=\frac{\varepsilon_{u}}{\varepsilon_{m}}\times 100$$ (2)

MSPU itself exhibits good shape memory behavior; both shape recovery and shape fixity are more than 90%. This shows that the hard segment plays a role as a fixed structure in memorizing the original shape quite well (Figure 6a and Table 4) [5].

Shape recovery is improved by PG, and further by MG in the composites prepared by the *in situ* method (Table 4). This shows that both PG and MG inhibit the permanent slip of hard segments, which causes the original shape to be forgotten. The interaction at the interface between graphene and SPU is a cause of this inhibition, and this interaction is more evident for MG, because it has many hydroxyl groups on the surface to make covalent bonds with SPU, although it scatters the well-ordered hydrogen bonds between the hard segments. In the case of graphene/SPU composites prepared by the physical mixing method or the MG/HSPU composites prepared by the *in situ* method, the shape recovery enhancement by graphene is marginal or negative (Table 4). Poor dispersion of graphene (Figure 2) in the composites and consequent ineffective interaction between graphene and SPU contribute to this result.

Table 5 shows that the shape recovery force is also improved by the reinforcing effect of graphene. However, the initial recovery forces are generally less than that of the tensile stresses at 100% elongation (Table 3). Recovery forces further reduce after one hour. This shows that stress relaxation due to irrecoverable rearrangements of SPU chains or of dispersed graphenes have occurred during elongation as well as during the second heating at 60 °C at fixed 100% strain. The recovery forces of MSPU-S-MG10 are better than those of MSPU-S-PG10 or MSPU-P-MG10 (Table 5), although the tensile stress at 100% elongation of MSPU-S-MG10 is lower than that of MSPU-S-PG10 or MSPU-P-MG10 (Table 3). These results show that the stress relaxation can be minimized by the grafting of SPU to graphene because it can reduce the slippage by the imposed external force of SPU chains adhered to graphene.

**Figure 7.**
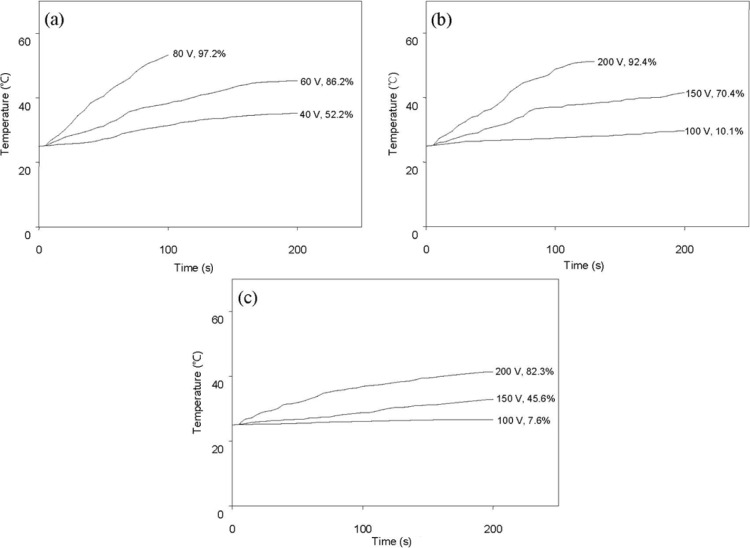
Surface temperature change and shape recovery of graphene/SPU composites by resistive heating: (**a**) MSPU-S-PG20; (**b**) MSPU-S-MG20; and (**c**) HSPU-S-MG20.

Figure 7 shows the variation of surface temperature over time following AC power application and shape recovery (both indicated at the end of each curve) due to resistive heating. Here, shape recovery is defined by Equation (1). In this experiment, composites containing 0.5 phr or 1 phr of graphene were not effectively actuated by an applied voltage up to 200 V. Figure 7 demonstrates that resistive heating and consequent shape recovery are more effective for composites having higher conductivity (Table 1).

**Figure 8.**
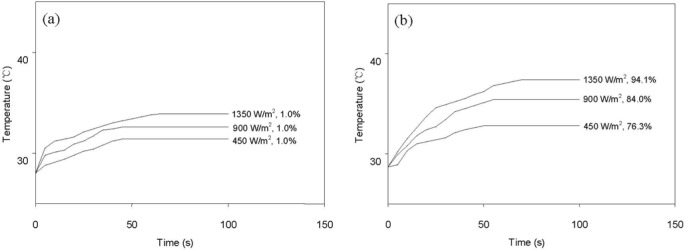
Surface temperature change and shape recovery of graphene/SPU composites by IR heating: (**a**) HSPU and (**b**) HSPU-S-MG05.

Figure 8 shows the variation of surface temperature and shape recovery following IR heating at various light intensities. The pristine HSPU recovered its original shape marginally (Figure 8a) regardless of light intensity. In contrast, HSPU-S-MG05 recovered considerably (Figure 8b), although the apparent surface temperature is similar to Figure 8a. This shows that finely dispersed MG effectively transfers the light energy to every part of the composite, even though the loading was as low as 0.5 phr. The maximum surface temperature and the shape recovery after 100 s caused by IR illumination, 900 W/m^2^ on the sample surface, are summarized in Table 6. These results show that IR heating efficiency was dependent on the amount and the kind of graphene, but dependence on the kind of SPU or the preparation method of the composite was marginal. MG exhibits better efficiency than PG. The graphitic structure of the *sp*^2^ carbon network, and the IR absorption bands of the C–O bond around 1230 cm^−1^ and of the O–H bond around 3430 cm^−1^ were strengthened by modification with MeOH [21]. This is the reason for the enhanced efficiency of IR heating for MG [34,35].

## 3. Experimental Details

### 3.1. Materials

Expandable graphite (ES350 F5, average particle size: 280 μm) purchased from Qindao Krompfmuehl Graphite Co., Ltd., (Qindao, China) was used for the preparation of graphene. The PCL diol (8000 g/mol; Solvay Chemicals, Houston, TX, USA) was dried and degassed at 80 °C under vacuum for 3 h. The MDI (BASF, Ludwigshafen, Germany), HDI (Nippon Polyurethane, Tokyo, Japan), 1,4-butanediol (BD; BASF, Ludwigshafen, Germany), *di*-*n*-octyltin dilaurate (DOT; Songwon Industrial Co., Ltd., Ulsan, Korea), and methyl ethyl ketone (MEK; Aldrich, St. Louis, MO, USA) were used as received.

Graphene was produced by rapid heating (at 1100 °C for 1 min under N_2_ atmosphere) of dry graphite oxide, which was prepared by the chemical oxidation of graphite using the Brodie method, as described in previous papers [20–22]. The graphene was modified with methanol to increase the number of hydroxyl functional groups on the graphene sheet. This increase was due to the reaction of epoxy groups on the graphene sheet with methanol, as reported in our previous paper [21]. The typical characteristics of the PG and the MG are shown in Table 7. More information about PG and MG is provided in our previous papers [21,22,36]. The FTIR spectrum and other data demonstrated that MG had more hydroxyl groups than PG [21].

### 3.2. Preparation of Graphene/SPU Composite

A 500 mL round-bottom, four-necked separable flask was equipped with a mechanical stirrer, a nitrogen inlet, a thermometer, and a condenser with a drying tube. To prepare the graphene/SPU composite by an *in situ* polymerization method, the graphene was immersed in MEK and then sonicated for 1 h. This resulting sonicated graphene mixture was approximately 1% solid content by weight. It was mixed with PCL diol (0.326 mol) in a round-bottom reactor and agitated at 80 °C for 1 h. Excess MEK was removed by evaporation during this agitation. This mixture was reacted with HDI (2.000 mol) in the presence of DOT (0.03 parts per 100 parts of total solids) for 2 h at 80 °C under dry N_2_ atmosphere. BD (1.674 mol) was then added and the reaction continued for another 3 h at 80 °C. As the reaction progressed, the increasing viscosity of the mixture was controlled by the addition of MEK. Upon completion of the reaction, the solid content of the polymer solution was about 45 wt%. When MDI was used as a diisocyanate instead of HDI, MDI was reacted with PCL diol at 80 °C for 2 h, and then 3 h at 80 °C with BD in the absence of DOT. The recipe for the preparation of SPU was designed so that the content of the soft PCL segment in the SPUs was always 80 wt%. The average molecular weights of the HSPU and the MSPU prepared in the absence of graphene, as measured by gel permeation chromatography using polystyrene standards, were 47,000 and 50,000, respectively.

To prepare the graphene/SPU composite using a physical mixing method, graphene was dispersed in MEK and sonicated for 1 h. This sonicated mixture, which contained approximately 1 wt% of graphene, was fed into the 30 wt% SPU solution in DMF and agitated at 80 °C for 3 h to obtain the graphene dispersion mixed with the SPU solution.

Composite films were cast on polypropylene plates at 25 °C for 24 h, and then at 60 °C for 24 h. The sample designation codes in Table 2 provide information regarding the kind of SPU, the preparation method, and the kind and the amount of the graphene used for the composite preparation. For example, MSPU-S-PG05 is the composite prepared with 100 parts of MSPU and 0.5 parts of PG (PG05) by the *in situ* method (S). HSPU-P-MG10 is the composite prepared with 100 parts of HSPU and 1.0 part of MG (MG10) by the physical mixing method (P).

### 3.3. Measurements

The TGA was performed (Mettler Toledo, SDTA 851e, Greifensee, Switzerland) to measure the residual weight after thermal degradation at a heating rate of 10 °C/min under a N_2_ atmosphere with 22 mg of sample in a platinum crucible. The morphology of a 10-μm-thick composite film was imaged using a Nikon (Tokyo, Japan) Eclipse LV100 optical microscope equipped with an Artcam-300MI-DS digital camera. The cryogenically fractured surface of the composite was observed using a field emission scanning electron microscope (JEOL, JSM-6500F, Tokyo, Japan). The direct current conductivity across a 0.5 mm-thick composite film was measured with a picoamperometer (Keithley 237, Cleveland, OH, USA) at room temperature using round silver electrodes of 0.28 cm^2^ that were attached to both surfaces of the specimen. Silver paste was used to ensure good contact between the specimen and the electrodes.

The DSC was carried out using a TA Instrument (New Castle, PA, USA) Q10 at a heating and cooling rate of 20 °C/min with 7 mg of sample. After loading at room temperature, the sample was heated up to 210 °C. Afterwards, it was cooled down to −80 °C to measure its crystallization temperature (*T_c_*) and heat of crystallization (*ΔH_c_*), and then heated up to 210 °C to measure the melting temperature (*T_m_*) and heat of fusion (*ΔH_m_*).

The FTIR spectra were recorded using an FTS 2000 FTIR (Varian, Palo Alto, CA, USA) employing a KBr tablet that was made by compression molding of the KBr powder mixed with a small amount of sample. The density of the cast composite film was calculated by dividing its weight by its volume. The volume of the composite was estimated from the volume change observed when the cast film was immersed in 25 °C water.

Tensile properties at 60 °C were examined with a tensile tester (OTU-2, Oriental TM Co., Siheung, Korea). The cast composite film was cut into a micro-tensile specimen 20 mm in length, 9 mm in width, and 0.5 mm in thickness. The specimen was elongated at a rate of 100 mm/min. Shape memory behaviors triggered by direct heating were also examined with the same tensile tester using samples of the same size. The cyclic tests to examine the shape memory effect of composite films are shown in Figure 6b. Samples were elongated in their rubbery state at a temperature above the *T_m_*, 60 °C to 100% strain (ε*_m_*) at a constant elongation rate of 100 mm/min. While maintaining the strain at ε*_m_*, the sample was cooled to a temperature below the *T_m_*, 25 °C, and then unloaded. Upon removing the constraint at 25 °C, a small recovery of strain to ε*_u_* occurred. The sample was subsequently heated again to 60 °C and, on leaving it freely at that temperature for the next 10 min, it was allowed to recover to the original shape. This process completed one thermomechanical cycle (*N* = 1), producing a residual strain of ε*_p_* at which position the next cycle (*N* = 2) began [5,21,22]. The shape recovery force was measured as follows: The sample was elongated from 60 °C to 100% strain at a constant elongation rate of 100 mm/min and then cooled to 25 °C, while holding the 100% strain with specimen clamps at both ends. The sample was heated again to 60 °C, keeping the 100% strain, to measure initial recovery force and that after one hour [10].

In order to evaluate the effectiveness of graphenes in the shape memory actuation of graphene/SPU composites by resistive heating, composite films 40 mm long, 9 mm wide, and 0.5 mm thick, were elongated at 60 °C to have a strain of 100% with a constant elongation rate of 100 mm/min. After this process, they were quickly cooled to 25 °C in their deformed states. Both ends of this deformed sample were clamped with conductive jaws, and an AC power of 60 Hz was applied through these jaws. A digital thermometer was used to measure the surface temperature with a thermocouple in contact with the sample [22].

For actuation by heating with IR light, an Hg-Xe lamp, which emits IR light over a broad band of the spectrum from 600 to 1000 nm, was positioned 2 cm from the sample and was used as a light source. Light having a wavelength less than 600 nm was blocked by a filter. The light intensity illuminating the sample was adjusted to either 450, 900, or 1350 W/m^2^. The composite films, which were 40 mm long, 9 mm wide, and 0.5 mm thick, were elongated at 60 °C to have a strain of 100% with a constant elongation rate of 100 mm/min. They were then quickly cooled to 25 °C in their deformed states. The sample, suspended in the air freely at room temperature, was illuminated for actuation by heating with IR light, and a digital thermometer was used to measure the surface temperature with a thermocouple in contact with the sample.

## 4. Conclusions

The compatibility of graphene with MSPU is better than that with HSPU, because both graphene and MDI-based MSPU have aromatic structures, whereas HDI, which is used for the preparation of HSPU, is aliphatic. The finely dispersed graphene enhanced the crystallization of the soft PCL segment in MSPU. However, poorly dispersed graphene in HSPU hindered the crystallization. The graphene in MSPU reinforced the MSPU and improved the shape recovery by direct heating more effectively than the graphene in HSPU. In addition, shape recovery by resistive heating of graphene/MSPU composites was better than that of graphene/HSPU composites, because of higher conductivity.

When graphene was modified with MeOH to increase the hydroxyl groups, the compatibility was further improved due to the enhanced compatibility by grafted SPU on graphene. However, conductivity of the composite, and thus the resistive heating efficiency, became worse because the grafted SPU hindered the intimate contact between the graphenes dispersed in SPU. The grafting of SPU on graphene improved shape recovery because it augmented fixed structure memorizing of the original shape. However the intimately mixed graphene disturbed the hydrogen bonds between hard segments and the dense packing of SPU. These phenomena offset the reinforcing effect of graphene at high graphene content.

For shape recovery actuated by IR heating, graphene modified with MeOH was more effective than PG, because the IR absorption bands of graphene were strengthened by modification with MeOH.

## Figures and Tables

**Table 1.** Characteristics of graphene/shape memory polyurethane (SPU) composites.

Sample	Conductivity (S/cm)	Density (g/cm^3^)
MSPU	(2.5 ± 0.5) 00D7 10^−11^	1.41 ± 0.13
MSPU-S-PG05	(1.7 ± 0.3) × 10^−7^	1.29 ± 0.13
MSPU-S-PG10	(1.6 ± 0.3) × 10^−4^	1.24 ± 0.12
MSPU-S-PG20	(4.6 ± 0.8) × 10^−3^	1.20 ± 0.08
MSPU-P-PG10	(6.9 ± 1.1) × 10^−4^	1.35 ± 0.14

MSPU-S-MG05	(4.0 ± 0.5) × 10^−11^	1.23 ± 0.11
MSPU-S-MG10	(5.9 ± 0.6) × 10^−5^	1.18 ± 0.10
MSPU-S-MG20	(1.9 ± 0.2) × 10^−4^	1.17 ± 0.12
MSPU-P-MG10	(5.1 ± 0.5) × 10^−4^	1.31 ± 0.14

HSPU	(6.0 ± 0.9) × 10^−11^	1.43 ± 0.15
HSPU-S-MG05	(2.4 ± 0.3) × 10^−10^	1.24 ± 0.10
HSPU-S-MG10	(2.3 ± 0.6) × 10^−6^	1.18 ± 0.08
HSPU-S-MG20	(2.8 ± 0.8) × 10^−6^	1.17 ± 0.09
HSPU-P-MG10	(2.4 ± 0.5) × 10^−5^	1.34 ± 0.05

**Table 2.** Thermal properties of graphene/SPU composites.

Sample	Thermal properties							
	Soft segment				Hard segment			
	*T_cs_*	*T_ms_*	Δ*H_cs_*	Δ*H_ms_*	*T_ch_*	*T_mh_*	Δ*H_ch_*	Δ*H_mh_*

	(°C)	(°C)	(J/g)	(J/g)	(°C)	(°C)	(J/g)	(J/g)
MSPU	16.0	52.4	35.2	38.6	–	–	–	–
MSPU-S-PG05	22.5	52.2	35.8	39.2	–	–	–	–
MSPU-S-PG10	22.7	51.9	37.5	43.5	–	–	–	–
MSPU-S-PG20	23.2	53.2	37.3	42.4	–	–	–	–
MSPU-P-PG10	15.5	51.6	29.8	32.7	–	–	–	–

MSPU-S-MG05	23.5	52.8	40.2	43.0	–	–	–	–
MSPU-S-MG10	23.7	54.0	36.5	38.8	–	–	–	–
MSPU-S-MG20	16.9	53.1	28.0	33.6	–	–	–	–
MSPU-P-MG10	18.1	51.2	39.3	44.8	–	–	–	–

HSPU	24.7	54.2	35.4	39.5	138.4	164.3	8.8	9.7
HSPU-S-MG05	19.4	53.7	34.7	34.0	134.0	168.0	3.9	6.9
HSPU-S-MG10	16.9	52.7	31.7	33.1	110.4	153.0	2.5	4.0
HSPU-S-MG20	20.1	54.9	32.6	32.6	–	158.2	–	3.0
HSPU-P-MG10	22.3	55.6	35.7	34.7	134.6	163.9	5.5	5.3

**Table 3.** Tensile properties of graphene/SPU composites.

Sample	Secant modulus at 100% elongation (MPa)	Tensile strength (MPa)	Elongation at break (%)
MSPU	0.59 ± 0.13	2.73 ± 0.26	551 ± 11
MSPU-S-PG05	1.52 ± 0.26	4.80 ± 0.41	625 ± 21
MSPU-S-PG10	1.79 ± 0.24	3.15 ± 0.35	363 ± 8
MSPU-S-PG20	3.35 ± 0.42	5.84 ± 0.42	332 ± 8
MSPU-P-PG10	1.89 ± 0.28	3.46 ± 0.33	461 ± 10
MSPU-S-MG05	1.73 ± 0.34	4.49 ± 0.45	606 ± 18
MSPU-S-MG10	1.63 ± 0.12	2.88 ± 0.24	344 ± 7
MSPU-S-MG20	1.69 ± 0.21	2.96 ± 0.26	90 ± 3
MSPU-P-MG10	2.10 ± 0.40	5.05 ± 0.81	425 ± 6
HSPU	2.48 ± 0.33	7.54 ± 0.94	615 ± 20
HSPU-S-MG05	4.65 ± 0.51	4.16 ± 0.58	194 ± 4
HSPU-S-MG20	4.12 ± 0.32	3.82 ± 0.62	218 ± 6
HSPU-P-MG10	2.00 ± 0.26	6.56 ± 0.94	577 ± 14

**Table 4.** Shape memory properties of graphene/SPU composites triggered by direct heating.

Sample	Shape recovery (%)				Shape fixity (%)			
	*N* = 1	*N* = 2	*N* = 3	*N* = 4	*N* = 1	*N* = 2	*N* = 3	*N* = 4
MSPU	97.5	96.2	94.1	92.2	98.7	97.4	96.1	95.9
MSPU-S-PG05	98.3	97.2	96.1	94.9	98.9	98.2	97.4	96.8
MSPU-S-PG10	97.4	96.3	95.0	93.7	99.9	99.6	99.0	98.6
MSPU-S-PG20	97.9	97.8	97.3	96.6	98.0	97.9	97.2	96.9
MSPU-P-PG10	96.6	95.7	94.9	94.2	99.3	99.2	98.8	98.2

MSPU-S-MG05	98.5	97.2	96.7	96.5	99.1	98.2	97.4	96.8
MSPU-S-MG10	98.5	97.8	97.7	96.2	99.1	98.7	97.9	97.1
MSPU-S-MG20	99.6	99.3	98.7	97.9	99.6	99.2	98.9	98.5
MSPU-P-MG10	94.3	93.4	91.3	89.2	99.4	98.6	97.9	97.4

HSPU	95.4	94.3	93.9	93.6	97.2	96.4	95.9	95.2
HSPU-S-MG05	96.5	95.6	93.8	92.0	98.5	98.0	97.2	96.3
HSPU-S-MG20	94.9	91.5	91.1	90.9	99.1	98.4	97.4	96.7
HSPU-P-MG10	93.9	93.1	92.1	91.6	97.2	96.4	95.2	94.8

**Table 5.** Recovery forces of graphene/SPU composites.

Sample	Recovery force (MPa)	
	Initial	After 1 h
MSPU	0.42 ± 0.08	0.25 ± 0.03
MSPU-S-PG05	0.73 ± 0.19	0.37 ± 0.04
MSPU-S-PG10	0.78 ± 0.14	0.55 ± 0.06
MSPU-P-PG10	0.56 ± 0.12	0.39 ± 0.04
MSPU-S-MG05	0.86 ± 0.13	0.76 ± 0.16
MSPU-S-MG10	1.18 ± 0.15	0.86 ± 0.09
MSPU-P-MG10	0.78 ± 0.11	0.62 ± 0.11

**Table 6.** Shape recovery of graphene/SPU composites triggered by IR heating.

Sample	Maximum surface temperature (°C)	Shape recovery (%)
MSPU	30.0	1.0
MSPU-S-PG05	33.7	90.3
MSPU-S-PG10	35.8	94.4
MSPU-S-PG20	48.9	97.1
MSPU-P-PG10	35.1	91.9
MSPU-S-MG05	37.5	94.4
MSPU-S-MG10	41.2	96.7
MSPU-S-MG20	51.2	99.5
MSPU-P-MG10	40.9	95.2
HSPU	33.9	1.0
HSPU-S-MG05	37.4	94.1
HSPU-S-MG10	41.1	95.4
HSPU-S-MG20	50.8	96.9
HSPU-P-MG10	40.1	95.1

**Table 7.** Characteristics of graphenes.

Sample	Composition	Particle size (μm)			Conductivity(S/cm)
		D (*v*, 0.1)	D (*v*, 0.5)	D (*v*, 0.9) ^a^	
PG	C_10_O_0.38_H_0.78_	2.7	8.3	20.6	26.8 ± 1.6
MG	C_10_O_0.89_H_0.93_	2.9	8.7	21.1	21.5 ± 1.2

a 90% of the volume distribution is below this value. Pristine graphene (PG) and modified graphene (MG).
